# Causal Factors of Anxiety and Depression in College Students: Longitudinal Ecological Momentary Assessment and Causal Analysis Using Peter and Clark Momentary Conditional Independence

**DOI:** 10.2196/16684

**Published:** 2020-06-10

**Authors:** Jeremy F Huckins, Alex W DaSilva, Elin L Hedlund, Eilis I Murphy, Courtney Rogers, Weichen Wang, Mikio Obuchi, Paul E Holtzheimer, Dylan D Wagner, Andrew T Campbell

**Affiliations:** 1 Department of Psychological and Brain Sciences Dartmouth College Hanover, NH United States; 2 Department of Computer Science Dartmouth College Hanover, NH United States; 3 National Center for PTSD White River Junction, VT United States; 4 Department of Psychiatry Dartmouth-Hitchcock Medical Center Lebanon, NH United States; 5 Department of Psychology Ohio State University Columbus, OH United States

**Keywords:** depression, anxiety, self-esteem, stress, causality, ecological momentary assessments, mental health, network, college

## Abstract

**Background:**

Across college campuses, the prevalence of clinically relevant depression or anxiety is affecting more than 27% of the college population at some point between entry to college and graduation. Stress and self-esteem have both been hypothesized to contribute to depression and anxiety levels. Although contemporaneous relationships between these variables have been well-defined, the causal relationship between these mental health factors is not well understood, as frequent sampling can be invasive, and many of the current causal techniques are not well suited to investigate correlated variables.

**Objective:**

This study aims to characterize the causal and contemporaneous networks between these critical mental health factors in a cohort of first-year college students and then determine if observed results replicate in a second, distinct cohort.

**Methods:**

Ecological momentary assessments of depression, anxiety, stress, and self-esteem were obtained weekly from two cohorts of first-year college students for 40 weeks (1 academic year). We used the Peter and Clark Momentary Conditional Independence algorithm to identify the contemporaneous (t) and causal (t-1) network structures between these mental health metrics.

**Results:**

All reported results are significant at *P*<.001 unless otherwise stated. Depression was causally influenced by self-esteem (*t*-1 *r*_p_, cohort 1 [C1]=–0.082, cohort 2 [C2]=–0.095) and itself (*t*-1 *r*_p_, C1=0.388, C2=0.382) in both cohorts. Anxiety was causally influenced by stress (*t*-1 *r*_p_, C1=0.095, C2=0.104), self-esteem (*t*-1 *r*_p_, C1=–0.067, C2=–0.064, *P*=.002), and itself (*t*-1 *r*_p_, of C1=0.293, C2=0.339) in both cohorts. A causal link between anxiety and depression was observed in the first cohort (*t*-1 *r*_p_, C1=0.109) and only observed in the second cohort with a more liberal threshold (*t*-1 *r*_p_, C2=0.044, *P*=.03). Self-esteem was only causally influenced by itself (*t*-1 *r*_p_, C1=0.389, C2=0.393). Stress was only causally influenced by itself (*t*-1 *r*_p_, C1=0.248, C2=0.273). Anxiety had positive contemporaneous links to depression (*t* *r*_p_, C1=0.462, C2=0.444) and stress (*t* *r*_p_, C1=0.354, C2=0.358). Self-esteem had negative contemporaneous links to each of the other three mental health metrics, with the strongest negative relationship being stress (*t* *r*_p_, C1=–0.334, C2=–0.340), followed by depression (*t* *r*_p_, C1=–0.302, C2=–0.274) and anxiety (*t* *r*_p_, C1=–0.256, C2=–0.208). Depression had positive contemporaneous links to anxiety (previously mentioned) and stress (*t* *r*_p_, C1=0.250, C2=0.231).

**Conclusions:**

This paper is an initial attempt to describe the contemporaneous and causal relationships among these four mental health metrics in college students. We replicated previous research identifying concurrent relationships between these variables and extended them by identifying causal links among these metrics. These results provide support for the vulnerability model of depression and anxiety. Understanding how causal factors impact the evolution of these mental states over time may provide key information for targeted treatment or, perhaps more importantly, preventative interventions for individuals at risk for depression and anxiety.

## Introduction

### Depression and Anxiety Prevalence and Risk Factors

Worldwide, depression is estimated to affect over 300 million individuals, and anxiety is estimated to affect over 260 million individuals [1]. A recent survey in the United States showed that individuals 18-25 years of age had the highest rate of major depressive episodes with a prevalence of over 13% [2]. On college campuses across the United States, more than 66% of students reported overwhelming anxiety within the last 12 months and more than 46% reported being so depressed it was difficult to function [3]. In the same report, more than 27% of undergraduates were found to have been diagnosed or treated for anxiety, depression, or both. In this paper, we focus on first-year undergraduate students, as they are a high-risk demographic going through a variety of life changes, many of which are risk factors for depression and anxiety, including living in a new geographic location, attending a new school, and meeting new friends [4-6]. This complex and changing period in students’ lives provides a unique window into the complex relationship between mental health metrics.

Two factors that may precipitate anxious or depressive symptoms are increased stress and low self-esteem [4,7-10]. Because stress and low self-esteem are often correlated with anxiety and depression, it can be difficult to disentangle the causal associations between these factors. New methodological developments in causal network reconstruction permits the estimation of causal networks from time series data. Implementing these methods might allow for the estimation of the causal network relating stress, self-esteem, anxiety, and depression among college students. If increased stress and low self-esteem are causal markers that precede anxiety and depression, then these methods might be useful for earlier identification or preventative intervention for people at risk. Moreover, they may help to identify factors that could be targets for programmatic or institutional changes and, thereby, reduce the prevalence of anxiety and depression.

### Defining Mental Health Metrics for Ultrabrief Measurements

Identifying temporal patterns of occurrence and causal factors underlying these mental disorders will be critical to addressing the growing mental health problems on college campuses and elsewhere. An unresolved question is the exact nature of causal relationships between the four variables previously mentioned. Although multiple definitions exist for these constructs, we focus on a subset of these, so they can be surveyed frequently and with minimal effort needed by the subject. Stress can be defined as a negative emotional or physical experience that has subsequent physiological or behavioral changes directed toward adaptation, either by changing the situation or accommodating its effects [11]. In this study, we focus on self-reported stress, given the complexities associated with measuring actual stress exposure. State self-esteem is considered a person’s sense of their own worth or value at the current moment [12]. The Patient Health Questionnaire (PHQ)-4 is frequently used as a brief measure with relatively good diagnostic performance for depression and anxiety, being comprised of the PHQ-2 and Generalized Anxiety Disorder-2 (GAD-2) [13]. As measured within this context, anxiety will be considered persistent and excessive worry. Similarly, depression is multifaceted, but we will focus on anhedonia and negative affect components, given their gross diagnostic abilities of anxiety and depression in college students [14]. Although focusing on these specific aspects of these mental health metrics is likely to be limiting, they allow for quick and more frequent assessments while maintaining rough diagnostic abilities.

### Models of Depression and Anxiety

There are a variety of models that predict how these variables influence each other across the lifespan. Data supporting these models have generally been sparsely sampled, with weeks or months separating 3-5 waves (time points) for each survey [15,16]. Here we focus on a few of these models, giving precedence to models with strong empirical support where primary relationships between two or more variables are mentioned. The vulnerability model of mental health [17] suggests that vulnerability and the likelihood of a mental health episode are linked in a trait-state relationship. Recent adaptations of the vulnerability model specifically predict that low self-esteem will lead to subsequent increases in depression and have received strong empirical support [18]. The diathesis-stress model postulates that there is a wide constellation of factors that may predispose someone to depression or anxiety and that stress is a moderating variable. Many models of anxiety are also based on the vulnerability model. These models include a stressor or trigger that may lead to subsequent increases in anxious symptoms and progress to clinical levels of anxiety, depending on the context [19,20]. The scar model predicts that depressive periods may lead to future low self-esteem [21,22]. The reciprocal relation model posits that stress leads to future anxiety and anxiety leads to future stress, and low self-esteem leads to increased depression and depression leads to future low self-esteem [18]. These diverse and not always complementary models beg testing with many time points, which may be able to more fully elucidate the relationship between true causal relationships. At a time where replication in social and affective neuroscience is in question, it is critically important to replicate results in distinct cohorts when possible [23,24].

### Ecological Momentary Assessments to Measure Mental Health Metrics

Increasing the sampling rate and study duration compared to previous causal studies would provide unique insight into the relationships among these mental health metrics. Recent technological advances allow for more frequent sampling with ecological momentary assessments (EMAs), which can be less invasive and more naturalistic than typical laboratory-based studies if the sampling frequency is not burdensome. With EMAs, questions can be sent directly to a person’s smartphone as often as desired [25,26]. EMA frequency should be balanced to minimize demand on the subject while maximizing coverage of potentially rapidly changing responses related to underlying mental health metrics with consideration for subject retention over the entire study duration. In an attempt to retain individuals across their entire collegiate experience, we selected weekly EMA sampling.

### Causal and Contemporaneous Network Detection

The ability to sample individuals’ mental health metrics more frequently allows for the possibility of testing models and theories present in the psychological literature, as well as generating new ones. With larger amounts of data, more methods to analyze causal influences can be considered. Autoregressive models such as Granger causality can be used to assess aspects of causation in an observed system [27]. Notably, Granger causality is susceptible to high dimensionality and missing data, and most implementations are not able to dissociate causal influences from highly correlated variables in a reasonable manner. Given the correlation present between the measured mental health metrics, Granger causality is not a reasonable method to select. Luckily, recent advances in causal network detection methods allow for parsing large, correlated time series with missing data. Peter and Clark Momentary Conditional Independence (PCMCI) is a method that can reconstruct robust causal networks for time series data that contain some missing points (ie, nonresponses from students in this study) while dealing with high dimensionality and highly correlated variables more effectively than many other methods while still retaining a low false-positive rate [28,29].

Although many research groups have demonstrated that stress, self-esteem, anxiety, and depression are all correlated, an unresolved question is if there are causal influences between these variables. A variety of psychological models related to interactions of these mental health metrics have been proposed, but much of the extant literature does not have temporal frequency and the total number of time points required to perform many of the recently developed causality methods. In this study, we aim to characterize the causal network of relationships between stress, self-esteem, depression, and anxiety as assessed by EMAs collected through the smartphone app, StudentLife. We first test this in a sample of first-year college students using PCMCI, then retest the same analysis in a completely independent cohort of subjects. Subsequently, we attempt to determine which psychological models of anxiety and depression best fit the current results and discuss possible implications.

## Methods

### Participants

Participants were first-year undergraduate students with eligible smartphones recruited from the Dartmouth College community during their first academic term. The Committee for the Protection of Human Subjects at Dartmouth College approved this study. Each participant provided written informed consent in accordance with guidelines set by the committee and received their choice of either course credit or monetary compensation (US $10 per week of EMAs answered) for study participation [30]. A subsample of cohort 1 (C1) participants were included in a previous study [31]. Two cohorts, corresponding to subsequent class years, were included. C1 included 106 subjects (75 females, mean age at the beginning of the study 18.25 years, SD 0.63, range 18-22). One subject was removed from the study for having a phone incompatible with our data collection app, leaving C1 with 105 subjects. Cohort 2 (C2) included 114 subjects (75 females, mean age at the beginning of the study 18.12 years, SD 0.45, range 18-20). One subject withdrew within a week of starting the study, and their data was excluded from further analyses, leaving C2 with 113 subjects.

### Ecological Momentary Assessments and Data Processing

Brief surveys of depression, anxiety, stress, and state self-esteem were assessed for 40 weeks (1 academic year) using EMAs (Multimedia Appendix 1), a method to assess an individual’s mental state in a naturalistic setting outside of the laboratory [25]. StudentLife, an app for Android and iOS, was installed on each participant’s smartphone [32] and used to administer EMAs at a random time point once per week for 40 weeks. Individuals were also able to manually open the StudentLife app and take the EMA in an unprompted manner. Data from StudentLife is uploaded to a secure server whenever a participant is both using WiFi and charging their phone, which they were encouraged to do daily. The EMA questions analyzed in this study included the PHQ-4, with depressive (PHQ-2) and anxious (GAD-2) components [13]. State self-esteem was measured with three questions selected from the State Self-Esteem Scale, which included a relevant question from each of the following categories: social, appearance, and performance [33]. Stress was measured by asking “Are you feeling stressed now?” with a 5-point Likert scale [34] with response labels ranging from “Not at All” to “Extremely.” Anonymized data was exported from StudentLife servers. Responses for an individual were averaged in the rare case that multiple responses were received within a given week. Individuals were able to access and answer the EMAs whenever they wanted, not just when prompted by the app. Weekly responses from all subjects were concatenated and buffered with three lines of missing values so that transition points between subjects would not weigh as causal influences. All missing values were replaced with “999.” C1 had 3404 weeks of responses to all four mental health metrics (mean 28.8, median 30, SE 0.74) across 105 individuals, and C2 had 3614 weeks of responses (mean 26.7, median 29, SE 0.77) across 114 individuals. A total of 7018 out of 8760 possible weeks contained responses across 219 individuals (80% response rate).

### Causal and Contemporaneous Network Detection

The goal of the current work is to determine the causal and contemporaneous network structure among the mental health metrics collected on a weekly basis. Contemporaneous network structure is the relationship between variables within the same time point, while causal network structure is the relationship between variables at different time points. PCMCI has the advantage of being able to recover causal networks that include multiple sources of causation and is suitable for data sets with missing responses and correlated variables [28,29,35]. PCMCI was implemented using the partial correlation method from the Tigramite software package (version 4.0.0) [36]. PCMCI is a two-step method that begins with a fully connected causal network graph. Condition selection, or PC1, a modification of the Peter and Clark algorithm, is the first step of PCMCI, which attempts to reduce the number of connections in the graph. Momentary conditional independence (MCI) is the second step of PCMCI, which consists of testing links for causal relationships (using partial correlations in this study).

Condition selection is a method to reduce the number of network connections that are interrogated for causal influences. Condition selection identifies nodes from previous time points that are likely to be real connections. Condition selection is the first phase of the PCMCI algorithm implemented in Tigramite [37] and is performed to reduce the number of potential causal connections interrogated. It applies a fast variant of PC1, a modification of the Peter and Clark algorithm [38] (for an in-depth description of PC1 and PCMCI please refer to [29]). PC1 identifies relevant conditions (variable at a given time lag) that may have predictive power for all variables. In this context, condition selection is performed using a liberal initial alpha value of .2 to retain more possible connections than not (as is recommended by the software author in the documentation [36] and is the default for the function in Tigramite version 4.0.0) while reducing the total number of connections to be tested in the second phase of PCMCI.

MCI, the second step of PCMCI, identifies contemporaneous and causal relationships from the reduced set of connections passed to it from the PC1 algorithm. Contemporaneous relationships are those that occur at the same time or faster than the sampling rate (within the same week in this study) and directionality cannot be assessed. Causal relationships are those that occur across time points (between weeks in this study). MCI was implemented through partial correlation estimated with a linear ordinary least squares regression and a test for nonzero linear Pearson correlation on the residuals at an alpha of .005. By using condition selection (PC1) paired with MCI using the partial correlation test, this method is able to account for autocorrelation, leading to well-controlled false-positive rates [28]. Partial correlation was selected from available test statistics within this framework due to its ease of interpretability, ability to work with all survey values, and ability to measure the association between a given pair of variables while removing variance associated with other variables, critical when looking at highly correlated mental health metrics. Visualization of contemporaneous and causal network graphs were generated using qgraph [39].

## Results

**Figure 1:**
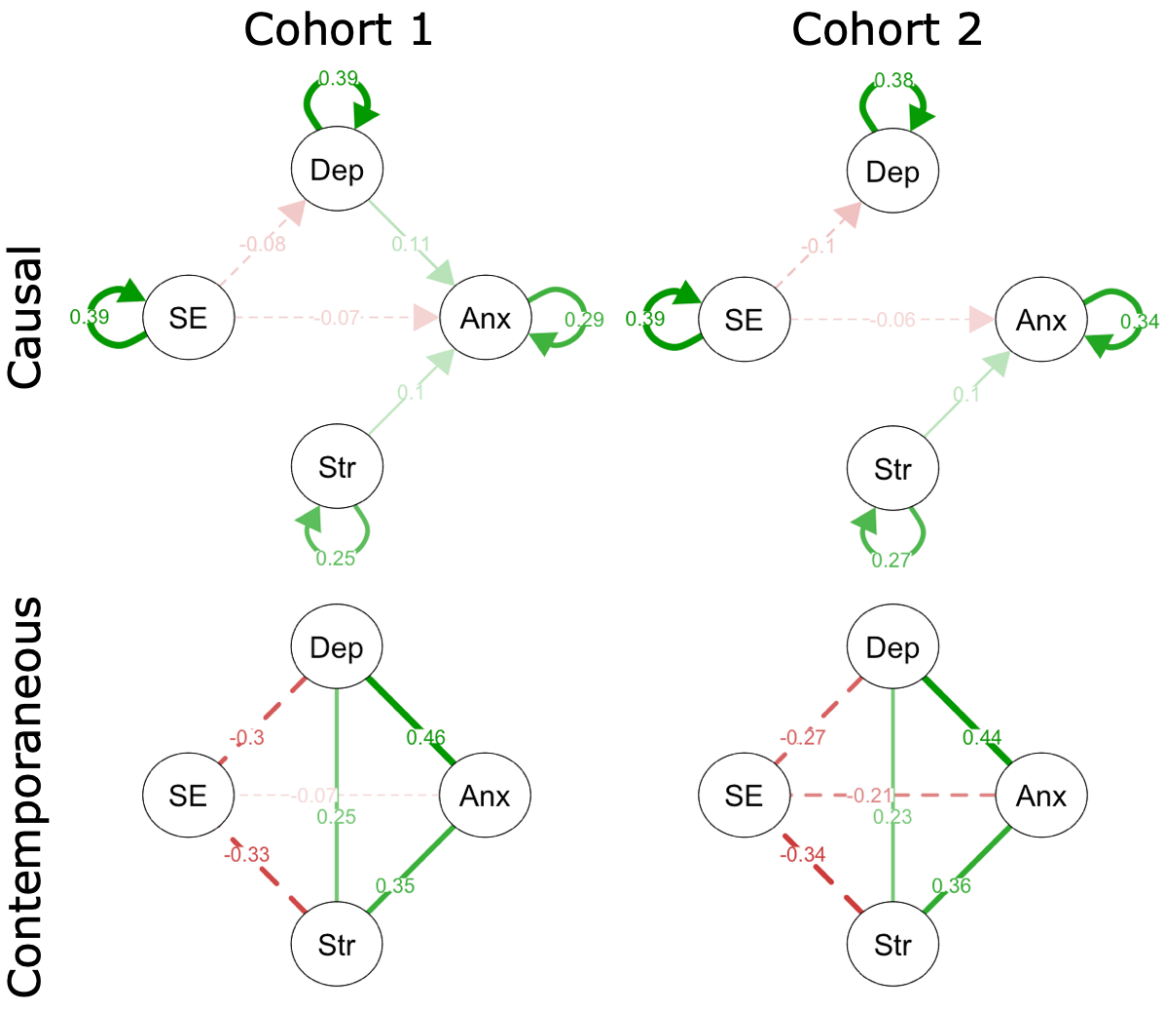
Causality graph observing previous week (t-1) connections on mental health metrics collected through ecological momentary assessments weekly for cohorts 1 (top left) and 2 (top right) at a threshold of *P*<.005. Contemporaneous connections presented for cohorts 1 (bottom left) and 2 (bottom right). Solid lines are positive relationships and dashed lines are negative relationships with magnitude marked on the graph, listing the partial correlation value. For causal relationships, the arrowhead signifies directionality. Anx: anxiety; Dep: depression; SE: self-esteem; Str: stress.

Causal influences are shown as directed lines with arrow heads revealing directionality (Figure 1, top), while contemporaneous influences are shown separately with undirected lines for clearer visualization (Figure 1, bottom). All displayed connections were significant at *P<.*005. Relationships described in the text were significant at *P*<.001 unless otherwise mentioned. Depression was causally influenced by self-esteem (*t*-1 *r*_p_, C1=–0.082, C2=–0.095) and itself (*t*-1 *r*_p_, C1=0.388, C2=0.382) in both cohorts. Anxiety was causally influenced by stress (*t-1* r_p_, C1=0.095, C2=0.104), self-esteem (*t*-1 *r*_p_, C1=–0.067, C2=–0.064, *P*=.002), and itself (*t*-1 *r*_p_, C1=0.293, C2=0.339) in both cohorts. A causal link between anxiety and depression was observed in the first cohort (*t*-1 *r*_p_, C1=0.109) and only observed in the second cohort with a more liberal threshold (*t*-1 *r*_p_, C2=0.044, *P=.*03 not displayed in Figure 1). Self-esteem was not causally influenced by any metrics except for itself (*t*-1 *r*_p_, C1=0.389, C2=0.393). Stress was not causally influenced by any metrics except for itself (*t*-1 *r*_p_, C1=0.248, C2=0.273).

The PCMCI partial correlation (*r*_p_) analysis of contemporaneous relationships revealed significant contemporaneous influences (*P<.*001) among all four mental health metrics (Figure 1, top). Observed contemporaneous undirected links (time *t*) included positive links between anxiety, depression, and stress. Anxiety had positive contemporaneous links to depression (*t* *r*_p_, C1=0.462, C2=0.444) and stress (*t* *r*_p_, C1=0.354, C2=0.358). Self-esteem had negative contemporaneous links to each of the other three mental health metrics, with the strongest negative relationship being stress (*t* *r*_p_, C1=–0.334, C2=–0.340), followed by depression (*t* *r*_p_, C1=–0.302, C2=–0.274) and anxiety (*t* *r*_p_, C1=–0.256, C2=–0.208). Depression had positive contemporaneous links to anxiety (previously mentioned) and stress (*t* *r*_p_, C1=0.250, C2=0.231).

## Discussion

### Summary of Primary Findings

Weekly EMAs of self-esteem, depression, stress, and anxiety were collected in two separate cohorts of over 200 college students across an academic year, with an average response rate of 80%. These EMAs were submitted to the PCMCI algorithm, which identified increased stress as a causal factor of future anxiety. Decreased self-esteem was identified as a causal factor of future depression and, to a lesser extent, anxiety. Increased depression was observed as a causal factor for future anxiety, but only in one of the two cohorts at a threshold of *P<.*005. These results help confirm the vulnerability model and extend previous literature by providing evidence for model testing.

This paper and previous research show congruent patterns of correlation among the mental health metrics observed [40]: positive relationships among depression, anxiety, and stress, and inverse relationships between self-esteem and the other mental health metrics. These findings are also congruent with previously validated similarities between these EMA surveys and longer web-based surveys of related mental health metrics [31,41]. Analyses using PCMCI provide insight into the causal temporal dynamics between these mental health metrics. Positive correlations were observed among depression, anxiety, and stress, while these 3 metrics were negatively correlated with self-esteem, as expected based on previous research.

### Causal Network Detection

The most novel and important part of this study is the application of the PCMCI algorithm for the identification of the causal network structure among mental health metrics [28]. PCMCI is a two-part algorithm, which when compared to other causality-detection algorithms, provides low false-positive rates combined with high power to detect real causal links. By reducing the number of connections tested in the causal network using PC1, PCMCI allows for higher dimensional data to be tested when compared with Granger causality or most other causal methods. By using partial correlation as the testing metric for the MCI phase of PCMCI, all variables are tested simultaneously, and interactions between correlated variables can be dissociated, something that has been elusive when implementing Granger causality. Within the current data set, conditioning with PC1 is likely less important, as we are using only four variables, while using partial correlation is critically important, and these mental health metrics are all at least somewhat correlated. The current results show that increases in stress cause subsequent increases in anxiety, while increases in self-esteem cause subsequent decreases in depression and, to a lesser extent, anxiety. These findings are consistent with the extant literature [7,40], but, to our knowledge, we are the first group to run a causal network analysis with moderately dense (weekly) temporal coverage extending across a whole academic year (40 weeks), identifying similar results across two distinct cohorts.

### Causal Findings and Existing Theories of Depression and Anxiety

The causal interactions among these mental health metrics can provide evidence to support some current psychological theories. This paper supports the vulnerability model, which suggests that low self-esteem can lead to subsequent depression or anxiety (Figure 1) [42,43]. We did not observe evidence supporting the scar model, where depression causally influences self-esteem, or the reciprocal relation model, where low self-esteem and depression have a feedback loop. Other groups have observed some support for the scar model, but to a lesser extent than the vulnerability model [7,8]. The meta-analysis by Sowislo and Orth [7] looked at prospective effects between these variables for each study, many of which included longer time frames with less frequent sampling than we observed here. It is possible that we would need to greatly increase the number of time points modeled as possible causal factors and collect data for a longer period of time to observe effects in support of the scar model.

The diathesis-stress model has been applied in both depressive and anxious contexts, where pre-existing conditions such as low self-esteem are risk factors, and stressful events can trigger subsequent increases in depression or anxiety in higher risk individuals [9]. There is some support for the diathesis-stress model in this paper, where self-esteem is a causal factor for anxiety and depression, and stress is a causal factor for anxiety. Conversely, with a time lag of 1 week, we did not see direct interactions between stress and self-esteem. It is possible that the interactions hypothesized between these variables in the diathesis-stress model may play out on a much longer timescale than observed here (multiple to many months or years, which has some support in the literature) or may primarily be related to trait self-esteem and not state self-esteem as measured here.

### Future Directions and Limitations

Although the current work provides an analysis of regularly sampled mental health metrics in a college population, a lag of 1 week was selected for simplicity and interpretability. It is possible that other lag intervals may provide additional insight into the causal dynamics of these variables. Given how rapidly mental states can change, weekly resolution may not be the optimal timescale to detect causality between these variables, where more frequent sampling could help further elucidate contemporaneous changes. Moreover, longitudinal sampling over many years may be better suited to testing the scar model [44]. There are a variety of moderating factors such as gender, socioeconomic status, and first-generation college student status that could potentially alter the observed results and should be investigated in future work.

The frequency of sampling needs to be balanced with the burden on study participants, since requesting frequent responses would likely lead to increased participant attrition and may even be an added stressor. As the current study looks to retain individuals over their entire college experience, minimizing the burden on the participant is likely to be a key to retention. Given the goal of long-term participant retention, the total number of questions asked through weekly EMAs was minimized, meaning limited coverage of anxiety or depressive symptoms. Although this may restrict the overarching implications that can be drawn from the current study, the questions were selected based on previous validation and maximal ability to diagnose anxious or depressive symptoms while asking minimal questions [13,14]. Furthermore, the questions from the PHQ-4 were not changed from Kroenke and colleagues [13], meaning they asked about the last 2 weeks, which may be suboptimal for the elucidation of causal factors and likely to underestimate the true magnitude of causal influences.

Values of these mental health metrics may change more rapidly than the weekly rate that they are sampled at in this study. Signals associated with fast fluctuations in these metrics would decrease our ability to identify relationships, particularly those that replicate across multiple cohorts, suggesting that the observed results are real and, if anything, underestimate the strength of the relationship between these variables. Increasing the sampling rate to daily or hourly could provide greatly improved insights into the nature of the temporal relationships between these metrics; although, it would increase participant burden and possibly decrease retention. A balance may be provided by using mobile smartphone sensing features to provide some insight into the optimal EMA sampling frequency for future studies. Smart triggering of EMAs, which is based on changes in smartphone features such as motion, conversation, or location, could reduce the total number of times a participant is asked to answer EMA questions while optimizing the temporal resolution and helping to better determine causal relationships among variables. Smartphone sensing features can be sampled hourly or even by the minute with little participant burden except for decreased battery life. Identifying sets of features such as the number of conversations, sleep, or number of locations visited [41] that predict shifts in mental states may help with both smart triggering of EMAs and identification of a more precise moment in time when an individual’s feelings change [45,46]. Identifying predictive features would add complexities to the data analysis pipeline and could be critical to balancing the amount of time requested of each participant over several years, as well as the temporal resolution needed to properly elucidate causal structures. Many theories and models have been created to describe the relationship among these mental health metrics. With increased survey sampling rates available with EMA and improvements in methods for detecting causal network structure, the ability to test these models and generate more refined ones becomes a possibility.

### Conclusions

This study provides a framework for identifying causal factors of anxiety and depression in college students, with the key results replicating in two distinct cohorts sampled weekly over the course of an academic year. Stress is a causal predictor of anxiety, while low self-esteem is a causal predictor of depression and, to a lesser extent, anxiety. These results support the vulnerability model of depression and suggest that ameliorating high rates of stress may reduce subsequent increases in self-reported anxiety. Continually testing and expanding models of the interactions between these and other mental health metrics will be critical to identifying causal factors and potential treatments or strategies to mitigate them, particularly in groups who are at a higher risk than the general population.

## Appendix

Multimedia Appendix 1 Ecological momentary assessments asked of participants on a weekly basis with the StudentLife app.

## Abbreviations

C1: cohort 1
C2: cohort 2
EMA: ecological momentary assessment
GAD-2: Generalized Anxiety Disorder-2
MCI: momentary conditional independence
PCMCI: Peter and Clark Momentary Conditional Independence
PHQ: Patient Health Questionnaire
