# Supplementary material for: Causal Factors of Anxiety and Depression in College Students: Longitudinal Ecological Momentary Assessment and Causal Analysis Using Peter and Clark Momentary Conditional Independence
Source: JMIR Ment Health. 2020 Jun 10;7(6):e16684. doi: 10.2196/16684 (PMC7315365; doi:10.2196/16684)
Supplement: Multimedia Appendix 1 [file mental_v7i6e16684_app1.doc]

| **Category** | **Question** | **Item** | **Response Options** |
| --- | --- | --- | --- |
| **Depression** | Over the last 2 weeks, how often have you been bothered by the following problems? | Little interest or pleasure in doing things | Not at all, Several days, More than half the days, Nearly every day |
|  |  | Feeling down, depressed, or hopeless |  |
| **Anxiety** | Over the last 2 weeks, how often have you been bothered by the following problems? | Feeling nervous, anxious or on edge |  |
|  |  | Not being able to stop or control worrying |  |
| **State Self-Esteem** | How much do you agree with this statement? | I am worried about what other people think of me. | Not at all, A little bit, Somewhat, Very much, Extremely |
|  |  | I am pleased with my appearance right now. |  |
|  |  | I feel as smart as others. |  |
| **Stress** | Are you feeling stressed now? |  |  |

Table S1. Ecological Momentary Assessments (EMAs) asked of participants on a weekly basis with the StudentLife app.
